# Effects of Omega‐3 Fatty Acid Supplementation on Serum Fetuin‐A Levels in Patients With Coronary Artery Disease

**DOI:** 10.1002/fsn3.70460

**Published:** 2025-06-18

**Authors:** Mehmet Arif Icer, Hilal Yıldıran, Asife Sahinarslan, Salih Topal

**Affiliations:** ^1^ Department of Nutrition and Dietetics, Faculty of Health Sciences Amasya University Amasya Turkey; ^2^ Department of Nutrition and Dietetics, Faculty of Health Sciences Gazi University Ankara Turkey; ^3^ Department of Cardiology, School of Medicine Gazi University Ankara Turkey

**Keywords:** anthropometric parameters, coronary artery disease, fetuin‐A, nutrient intake, omega‐3 fatty acid

## Abstract

Omega‐3 fatty acids (FAs) are being studied as potential regulators of serum fetuin‐A, linked to the risk of developing coronary artery disease (CAD). This study aims to assess the impact of omega‐3 FA supplementation on the level of serum fetuin‐A in patients with CAD. The research was conducted on a sample of 34 male participants between the ages of 35 and 75. These individuals had recently been diagnosed with CAD through the use of conventional coronary angiography. The patients diagnosed with CAD were separated into two groups: the “Omega‐3 Group” consisting of 16 individuals, and the “Control Group” consisting of 18 individuals. Both groups were provided with an explanation of the principles of a low‐fat diet at the beginning of the study. The patients in the omega‐3 group were administered a daily supplementation of 1.560 mg of omega‐3 FAs for 8 weeks, whereas the control group did not receive any supplementation. Food consumption was documented through the use of 6‐day dietary records. Fetuin‐A levels decreased significantly in both the omega‐3 and control groups from baseline to Week 8 (*p* < 0.001). Although there were no notable disparities in the initial serum fetuin‐A levels between the omega‐3 and control groups (*p* > 0.05), the analysis conducted at Week 8 revealed that the omega‐3 group exhibited higher serum fetuin‐A levels compared to the control group (*p* = 0.001). In addition, dietary protein (g) and animal protein (g) intakes correlated negatively with serum fetuin‐A levels in both groups, but not for omega‐3 FAs (g). These results suggest that there is no significant correlation between serum fetuin‐A levels and the amount of omega‐3 FAs taken in supplementation and/or diet, and dietary modifications may be an effective strategy to manage serum fetuin‐A levels.

**Trial Registration:** ClinicalTrials.gov NCT05661994

## Graphical abstract

It was found in the study that medical nutrition therapy (low‐fat diet principles) applied to patients with CAD with or without omega‐3 FA supplementation causes a decrease in serum fetuin‐A levels, and there was no significant correlation between serum fetuin‐A levels and the amount of omega‐3 FAs taken in supplementation and/or diet. In addition, study data suggest that dietary modifications may be an effective strategy to manage serum fetuin‐A levels. Due to the limited number of studies investigating the effects of omega‐3 fatty acid supplementation and nutritional status on serum fetuin‐A levels, it is thought that the results obtained from this study will guide future studies.

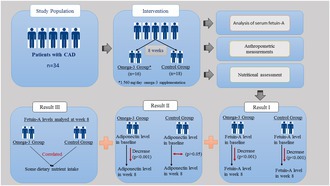

## 1 Introduction

Fetuin‐A, or Alpha 2‐Heremans‐Schmid Glycoprotein (AHSG), is a serum protein primarily produced by hepatocytes and subsequently released into the bloodstream (Bourebaba and Marycz 2019; Nawratil et al. 1996). Fetuin‐A is classified as both a hepatokine and an adipokine because it is secreted by both hepatocytes and adipocytes (Eswar et al. 2024).

In our previous publication, we highlighted that fetuin‐A is a multifunctional glycoprotein involved in various physiological and pathological processes, including diabetes, obesity, kidney diseases, coronary artery disease (CAD), tumor progression, and vascular calcification (Icer and Yıldıran 2020a). In one of our recent studies, we demonstrated that elevated serum fetuin‐A levels may serve as a risk factor for the development of obesity (Icer, Koçak, Ağagündüz, et al. 2025). Conversely, in another study, we found that decreased serum and urinary fetuin‐A levels may be associated with an increased risk of kidney stone formation (Icer, Koçak, Icer, et al. 2025). Although fetuin‐A is a multifunctional protein, its role in some diseases such as CAD has been studied more (Lin et al. 2024; Mohamed et al. 2024; Mori et al. 2024). It is thought that fetuin‐A may be effective in the regulation of vascular calcification with its different roles such as accumulation of calcium phosphate crystals (Mori et al. 2024) and annexin A2 fetuin‐A signaling (Icer and Yıldıran 2020a; Mancio et al. 2020). Contrary to previous research suggesting that elevated serum fetuin‐A levels are associated with an increased risk of CAD (Afrisham et al. 2021; Mehrotra et al. 2005), other studies have reported that low fetuin‐A levels may also be a risk factor (El‐Shishtawy et al. 2024; Lin et al. 2024; Mohamed et al. 2024; Mori et al. 2024), and that there is no clear association between fetuin‐A levels and CAD (Aroner et al. 2016; Mikami et al. 2008).

Dietary sources of omega‐3 FAs and fish oil consumption are inversely associated with cardiovascular disease (CVD) risk factors (Yan et al. 2024). Omega‐3 fatty acids (FAs) are thought to exert their potential protective effects against CVD through their influence on lipid metabolism, thrombosis, and inflammation (Bäck 2017; Kromhout et al. 2012; Mozaffarian and Wu 2011). However, the exact mechanisms by which omega‐3 FAs confer protection against CAD remain incompletely understood. One of the potential mechanisms under investigation is whether one of the mechanisms underlying this protective effect of omega‐3 FAs against CVD is through a modulatory effect on serum fetuin‐A levels. An exploration into the potential impact of omega‐3 FA supplementation on serum fetuin‐A levels linked to CAD will provide a more comprehensive comprehension of the interplay between omega‐3 FAs and CAD. It is thought that omega‐3 FA supplementation may provide clinical benefit in preventing vascular calcification and CVD by activating vitamin D, increasing fetuin‐A levels, and changing erythrocyte membrane FA content in dialysis patients (An et al. 2012). In our previous article investigating the effects of nutritional status on serum fetuin‐A levels, we emphasized the association between certain dietary parameters and serum fetuin‐A concentrations (Icer and Yıldıran 2020b). Several studies have reported conflicting findings regarding the impact of omega‐3 FA supplementation on serum fetuin‐A levels. Some studies have demonstrated an increase in serum fetuin‐A levels with omega‐3 FA supplementation (An et al. 2012; Werida et al. 2021). However, other studies have shown a decrease in serum fetuin‐A levels (Ozyazgan et al. 2013) or have found no significant effect (Khodadustan et al. 2021). Further studies are required to confirm the effect of omega‐3 FAs on serum fetuin‐A levels.

The limited research evaluating the impact of omega‐3 FA supplementation on serum fetuin‐A levels in patients with CAD underscores the importance of conducting such a study. This research contributes meaningfully to the existing literature by exploring whether the potential cardioprotective effects of omega‐3 FAs may be mediated through changes in serum fetuin‐A levels. The primary objective of the study is to assess the effect of omega‐3 FA supplementation on serum fetuin‐A concentrations in patients with CAD. Additionally, a secondary aim is to investigate the relationship between nutritional status, dietary habits, and serum fetuin‐A levels.

## 2 Materials and Methods

### 2.1 Study Design and Participants

We conducted a randomized, controlled, open‐intervention study between December 18, 2020 and October 11, 2021 on 34 male participants aged between 35 and 75 years old and had recently diagnosed CAD by conventional coronary angiography. The sample size was estimated assuming a 5% alpha error (i.e., the confidence level = 95%) and 80% power. Based on the difference between independent means, the minimum sample size was 15 participants per group. The software used for the sample size was based on G*Power 3.0.10. A post hoc power analysis was conducted using G*Power 3.1 to evaluate whether the sample size used in the paired samples *t*‐test was sufficient. Given an observed effect size of dz. = 0.571, an alpha level of 0.05, and a total sample size of 34, the achieved statistical power was calculated to be 0.947. This result indicates that the sample size was adequate to detect the observed effect with a high degree of power, exceeding the commonly recommended threshold of 0.80.

The study excluded individuals who had engaged in dieting within the past 6 months, were currently taking omega‐3 FAs and/or vitamin/mineral supplements, were prescribed statin group drugs, had a body mass index (BMI) below 18.5 or above 40 kg/m^2^, had a fasting blood glucose level of 126 mg/dL or higher, had systemic diseases such as Type 1 or Type 2 diabetes, kidney diseases, liver diseases, cancer, or neurological diseases, were active athletes, or were regular exercisers. The study was approved by the Clinical Research Ethics Committee of Gazi University Faculty of Medicine on 26 November 2020, with approval number 810. Furthermore, the Turkish Medicines and Medical Devices Agency granted approval under decision number 66175679‐514.11.01‐E.290378. The study was registered at clinicaltrials.gov with the identifier NCT05661994. Each participant provided written informed consent, and the study was carried out in compliance with the Helsinki Declaration.

**FIGURE 1:**
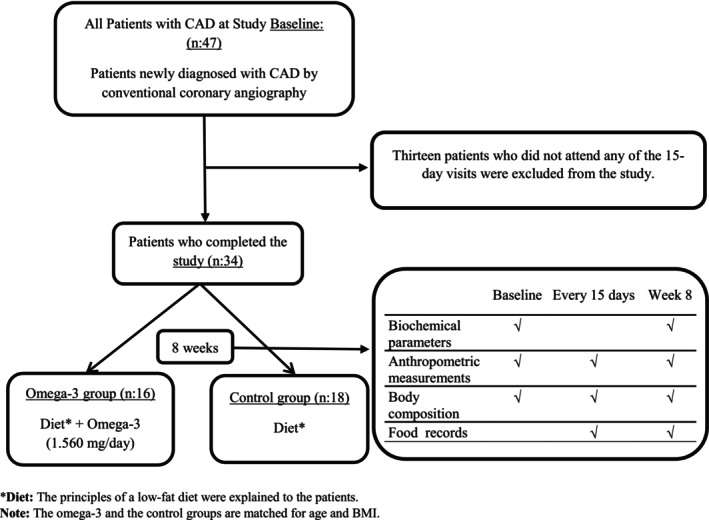
Flow diagram representing case selection for the trial program. A total of 34 patients with newly diagnosed coronary artery disease (CAD), confirmed by conventional coronary angiography, were enrolled and divided into two groups: the omega‐3 group (*n* = 16) and the control group (*n* = 18). The omega‐3 group received a dietary intervention along with omega‐3 fatty acid supplementation (1560 mg/day) for 8 weeks, while the control group received only the dietary intervention. CAD, coronary artery disease.

The study involved patients diagnosed with CAD who were divided into two groups: the “Omega‐3 Group” consisting of 16 individuals, and the “Control Group” consisting of 18 individuals. In the present study, the groups were matched for age, gender, and BMI. The purpose of the study was to assess the impact of omega‐3 FA supplementation on various biochemical parameters, with a particular focus on serum fetuin‐A levels, as well as anthropometric measurements and nutritional status. Sixteen participants with CAD were included in the omega‐3 group and received daily supplementation of 1.560 mg of omega‐3 FAs for 8 weeks. Additionally, they were provided with instructions on following a low‐fat diet. Eighteen participants with CAD in the control group were provided with an explanation of the principles of the low‐fat diet, without the addition of omega‐3 FA supplementation. Data from the participants were collected at the start, every fortnight, and at the conclusion of the eighth week. To assess participants' compliance with the interventions, 24‐h dietary recall interviews were conducted every 15 days throughout the 8‐week study period. During these assessments, adherence to the low‐fat dietary guidelines and regular intake of omega‐3 supplements was evaluated. Participants who failed to comply with the dietary recommendations and/or did not consistently take the omega‐3 supplements were excluded from the study based on these biweekly evaluations. The study design is depicted in Figure 1.

### 2.2 Omega‐3 Supplementation

One capsule of omega‐3 (Ocean Plus) used in the study contains 780 mg of omega‐3, including 384 mg of eicosapentaenoic acid (EPA), 252 mg of docosahexaenoic acid (DHA), and 144 mg of other omega‐3 FAs. By providing the patients in the omega‐3 group take 2 of these omega‐3 capsules a day, 1.560 mg/day omega‐3 supplementation was applied for 8 weeks. The amount of omega‐3 supplementation applied was decided in line with the recommendations of the American Heart Association (AHA) (Siscovick et al. 2017).

### 2.3 Low‐Fat Diet

In line with the AHA recommendations and DASH‐Mediterranean dietary principles, all patients in the study were explained a low‐fat diet to reduce the diet's total fat, saturated fat, and cholesterol content (American Heart Association 2022a, 2022b; D'Alessandro and De Pergola 2014; Mahan and Raymond 2016; National Institutes of Health 2006). These low‐fat diet principles explained without a specific energy restriction consist of three steps: recommendations (Eating fish, particularly the oily kind—twice a week, etc.), foods that should be limited in the diet (limiting added sugars to no more than 150 cal each day, etc.), and foods that are prohibited (offal, organ meats, etc.).

During the study, patient compliance with low‐fat diet principles was also questioned during the visits made every 15 days.

### 2.4 Nutritional Assessment

The researchers collected a total of 6‐day food records from the participants. This included 3‐day food records with a 24‐h dietary recall method every 15 days, as well as three consecutive‐day food records at Week 8. The purpose of collecting these records was to determine the participants' daily dietary energy and nutrient intake. For the determination of food quantities and the amounts included in the portions of the meals “Yemek ve Besin Fotoğraf Kataloğu” (Rakcolu et al. 2012) and “Kurumlar için Standart Yemek Tarifleri” was used (Merdol 2011). The energy and nutrient intake were determined utilizing the Nutrition Information System (BeBiS) Software (2006) (“BeBis (Beslenme Bilgi Sistemi) bilgisayar yazılım programı versiyon 7,”).

### 2.5 Anthropometric Measurements

The participants' body weight, body fat percentage, body muscle mass, and body water percentage were assessed using the Tanita bc 601 bio‐electric impedance device after a fasting period of 10–12 h. The BMIs were computed using the equation ‘body weight/height^2^ (kg/m^2^)’. The participants' height and waist circumferences were assessed using a non‐elastic tape measure.

### 2.6 Biyokimyasal Parametreler

The nurse collected blood samples from the participants after an 8‐h fasting period to analyze their biochemical parameters. The biochemistry laboratory of the Faculty of Medicine, Gazi University analyzed blood samples to determine the levels of total cholesterol (mg/dL), low‐density lipoprotein cholesterol (LDL‐C) (mg/dL), high‐density lipoprotein cholesterol (HDL‐C) (mg/dL), triglyceride (mg/dL), C‐reactive protein (CRP) (mg/L), fasting blood glucose (mg/dL), fasting insulin (μIU/mL), calcium (mg/dL), phosphorus (mg/dL), and high‐sensitivity troponin (T (hs) ‐TnT) (ng/mL) levels of the participants. Adiponectin (μg/mL) and fetuin‐A (ng/mL) concentrations in blood samples were analyzed in a private laboratory.

For the analysis of adiponectin and fetuin‐A levels, blood samples were collected in yellow‐capped gel biochemistry tubes and left upright at room temperature for 30–45 min. The samples were then centrifuged at 3000 rpm for 15 min at the Central Laboratory of Gazi University Faculty of Medicine Research and Application Hospital. The resulting serum was aliquoted into 1.5 mL Eppendorf tubes and stored at −80°C until analysis. Serum concentrations of fetuin‐A and adiponectin were measured using enzyme‐linked immunosorbent assay (ELISA) kits (Fetuin‐A: Elabscience Biotechnology Inc., Human Fetuin‐A ELISA Kit; Adiponectin: Elabscience Biotechnology Inc., Human ADP/Acrp30 (Adiponectin) ELISA Kit).

The Homeostatic model assessment‐insulin resistance (HOMA‐IR) values were computed using the formula: fasting insulin (mIU/mL) × fasting glucose (mg/dL)/405 (Bahadur et al. 2021).

### 2.7 Statistical Analysis

The data collected in this study were analyzed using IBM SPSS Statistics version 21. Qualitative variables were expressed as frequencies (*n*) and percentages (%), while quantitative data were summarized using means (X¯) and standard deviations (SD). The Kolmogorov–Smirnov test was used to assess the normality of data distribution. For categorical variables, associations between groups were evaluated using the chi‐square test. For normally distributed continuous data, the Independent Sample *t*‐test was used to compare two independent groups, and the Paired Sample *t*‐test was applied for comparisons within related (dependent) groups. For non‐normally distributed data, the Mann–Whitney *U* test was used for comparisons between independent groups, and the Wilcoxon signed‐rank test was employed for related (dependent) samples. Correlation analyses were conducted using the Pearson correlation coefficient for normally distributed variables, and the Spearman correlation coefficient when at least one variable did not meet the assumption of normality. Statistical significance was evaluated at confidence levels of 95% and 99%.

## 3 Results

There was no statistically significant difference between the omega‐3 and the control groups in terms of age, marital status, educational status, smoking status, amount of cigarettes smoked, and alcohol use (*p* > 0.05) (Table 1).

**TABLE 1:** Distribution of general characteristics of the patients.

	Omega‐3 group (*n*: 16)		Control group (*n*: 18)		*χ* ^2^	*p*
	*S*	%	*S*	%		
Marital status						
Married	13	81.3	17	94.4	1.421	0.233
Single	3	18.2	1	5.6		
Educational status						
Primary school	3	18.8	5	27.8	2.936	0.569
Middle School	1	6.2	4	22.2		
High school	4	25.0	2	11.1		
University	7	44.0	6	33.3		
Postgraduate	1	6.2	1	5.6		
Alcohol use						
Yes	2	12.5	2	11.1	0.016	0.900
No	14	87.5	16	88.9		
Smoking status						
Yes	5	31.2	10	55.6	2.030	0.154
No	11	68.8	8	44.4		
Amount of cigarettes smoked pcs/day $\left(\bar{X}\pm \mathrm{SD}\right)$	27.00 ± 19.87		17.00 ± 4.83		*p* = 0.144	
Average age (years) $\left(\bar{X}\pm \mathrm{SD}\right)$	56.12 ± 8.00		58.61 ± 7.84		*p* = 0.367	

*Note:* “Chi‐square” test was used to evaluate the relationship between categorical variables, and “Independent Sample *t*‐test” test was used to compare the measurement values of two independent groups with normal distribution.

Body weight (kg), BMI (kg/m^2^), waist circumference (cm), waist/height ratio, body fat percentage (%), body muscle mass (kg) and body water percentage (%) did not significantly change in both the omega‐3 and control groups from baseline to Week 8 (*p* > 0.05). The neck circumference decreased significantly in the omega‐3 group from baseline to Week 8 (*p* < 0.05), but not in the control group (Table 2).

**TABLE 2:** Anthropometric measurements, BMI and some body composition analyzes of the patients.

	Omega‐3 group (*n*: 16)				*p*	Control group (*n*: 18)				*p*	Group omega‐3‐control	
	Baseline		Week 8			Baseline		Week 8			Baseline	Week 8
	$\bar{X}$ ± SS	Alt‐Üst	$\bar{X}$ ± SS	Alt‐Üst		$\bar{X}$ ± SS	Alt‐Üst	$\bar{X}$ ± SS	Alt‐Üst		*p*	*p*
Body weight (kg)	88.28 ± 1183	71.80–114.00	87.88 ± 11.97	72.90–113.50	0.378	81.29 ± 15.95	60.60–120.00	81.61 ± 16.51	63.20–125.30	0.446	0.161	0.219
BMI (kg/m^2^)	29.07 ± 4.06	23.70–38.10	28.88 ± 3.94	24.06–37.96	0.189	27.64 ± 3.87	22.50–35.55	27.74 ± 4.00	22.20–36.20	0.486	0.302	0.410
Waist circumference (cm)	108.19 ± 9.97	93.00–126.00	107.31 ± 9.62	89.00–124.00	0.348	104.75 ± 12.30	87.00–136.00	105.56 ± 12.22	91.00–141.00	0.289	0.381	0.647
Waist/height ratio	0.62 ± 0.06	0.55–0.73	0.61 ± 0.05	0.53–0.72	0.337	0.61 ± 0.05	0.51–0.73	0.61 ± 0.05	0.54–0.76	0.111	0.657	0.996
Neck circumference (cm)	42.44 ± 3.61	37.00–49.00	41.84 ± 3.34	37.00–48.00	**0.037** *	41.22 ± 2.92	38.00‐47.00	41.03 ± 2.84	38.00–47.00	0.233	0.287	0.447
Body fat percentage (%)	25.67 ± 5.23	15.90–35.40	25.63 ± 3.82	18.50–32.90	0.959	24.39 ± 4.54	12.20–31.80	24.78 ± 5.09	13.50–34.80	0.613	0.452	0.593
Body muscle mass (kg)	62.14 ± 8.35	51.70–83.90	61.25 ± 7.71	51.20–77.00	0.327	60.26 ± 9.58	44.80–80.00	58.40 ± 9.92	44.50–86.90	0.252	0.548	0.361
Body water percentage (%)	53.33 ± 4.15	46.10–60.40	53.3 ± 2.85	46.50–58.20	0.966	54.32 ± 3.46	48.90–63.40	55.51 ± 5.77	46.20–71.60	0.347	0.463	0.237

*Note:* Independent Sample *t*‐test was used to compare the measurement values of two independent groups with normal distribution, while Paired Sample *t*‐test method was used to compare the measurement values of two dependent groups with normal distribution. In the data that did not have a normal distribution, the “Mann–Whitney *U*” test was used to compare the measurement values of two independent groups, and the “Wilcoxon signed‐rank” test was used to compare the measurement values of the two dependent groups. Bold indicates statistical significance rates were given 95% confidence interval for *p* < 0.05.

Abbreviation: BMI, body mass index.

* *p* < 0.05.

Fetuin‐A levels significantly decreased in both the omega‐3 and control groups from baseline to Week 8 (*p* < 0.001). While there were no significant differences in baseline serum fetuin‐A levels between the two groups (*p* > 0.05), the Week 8 analysis showed that the omega‐3 group had significantly higher serum fetuin‐A levels compared to the control group (*p* = 0.001) (Table 3).

**TABLE 3:** Serum fetuin‐A levels and some biochemical parameters of the patients.

Parameters	Omega‐3 group (*n*: 16)				*p*	Control group (*n*: 18)				*p*	Group omega‐3‐control	
	Baseline		Week 8			Baseline		Week 8			Baseline	Week 8
	$\bar{X}$ ± SS	Alt‐Üst	$\bar{X}$ ± SS	Alt‐Üst		$\bar{X}$ ± SS	Alt‐Üst	$\bar{X}$ ± SS	Alt‐Üst		*p*	*p*
Fetuin‐A (ng/mL)	1011.51 ± 69.97	885.93–1154.64	757.14 ± 50.12	650.62–833.51	**0.000** *	1006.82 ± 96.63	817.24‐1181.30	680.35 ± 69.85	508.61–785.79	**0.000** *	0.874	**0.001** *
Total cholesterol (mg/dL)	188.78 ± 47.92	91.40–246.00	140.13 ± 31.46	92.00–196.00	**0.000** *	211.67 ± 47.35	127.00‐281.00	143.83 ± 31.27	103.00–227.00	**0.000** *	0.171	0.733
LDL‐C (mg/dL)	124.94 ± 34.48	53.00–173.00	76.50 ± 24.80	41.00–122.00	**0.000** *	136.56 ± 36.53	86.00‐199.00	81.61 ± 30.16	47.00–177.00	**0.000** *	0.349	0.596
HDL‐C (mg/dL)	40.15 ± 10.91	21.00–61.00	37.69 ± 8.97	21.00–58.00	0.223	45.44 ± 8.48	30.00–59.00	42.11 ± 9.34	28.00–61.00	0.144	0.122	0.170
Triglyceride (mg/dL)	153.69 ± 90.06	46.00–418.00	127.13 ± 58.03	68.00–270.00	0.277	156.45 ± 86.66	57.00–373.00	124.11 ± 75.89	53.00–309.00	0.092	0.928	0.898
CRP (mg/L)	12.01 ± 19.36	0.77–79.50	9.42 ± 21.51	1.52–89.20	0.135	5.18 ± 5.44	1.00–21.51	4.01 ± 2.99	1.03–10.50	0.417	0.102	0.825
Fasting blood glucose (mg/dL)	106.31 ± 10.47	90.00–125.00	105.56 ± 12.68	89.00–124.00	0.825	97.06 ± 12.97	69.00–119.00	92.89 ± 11.91	74.00–116.00	0.181	0.780	**0.005** *
Fasting insulin (μIU/mL)	15.91 ± 17.62	3.67–74.64	16.32 ± 18.49	4.39–62.67	0.941	13.88 ± 20.59	1.58–84.25	9.00 ± 8.25	2.41–36.60	0.344	0.211	0.059
Adiponectin (μg/mL)	14.98 ± 5.47	4.69–27.16	19.89 ± 4.15	12.69–25.65	**0.001** *	20.80 ± 5.40	10.11‐27.44	19.60 ± 4.94	4.87–24.56	0.390	**0.004** *	0.853
Phosphorus (mg/dL)	3.16 ± 0.58	2.40–4.10	3.60 ± 0.45	2.90–4.30	**0.021** *	2.99 ± 0.69	2.00‐4.30	3.42 ± 0.68	2.20–4.80	**0.014** *	0.347	0.187
Calcium (mg/dL)	9.76 ± 0.32	9.30–10.50	10.03 ± 0.29	9.40–10.50	**0.017** *	9.45 ± 0.83	7.60‐11.00	9.74 ± 0.47	9.10–11.00	0.109	0.211	**0.011** *
hs‐TnT (ng/mL)	608.88 ± 1587.57	5.00–5646.00	12.38 ± 9.99	6.00–44.00	0.153	169.44 ± 374.23	5.00–1541.00	13.22 ± 11.27	5.00–49.00	0.088	0.313	0.646
HOMA‐IR	4.31 ± 5.37	1.09–23.04	4.33 ± 5.16	0.96–19.03	0.988	3.36 ± 4.85	0.34–18.93	2.09 ± 1.97	0.45–8.59	0.306	0.075	**0.025** *

*Note:* Independent Sample *t*‐test was used to compare the measurement values of two independent groups with normal distribution, while Paired Sample *t*‐test method was used to compare the measurement values of two dependent groups with normal distribution. In the data that did not have a normal distribution, the “Mann–Whitney *U*” test was used to compare the measurement values of two independent groups. Bold indicates statistical significance rates were given a 95% confidence interval for *p* < 0.05.

Abbreviations: CRP, C‐reactive protein; HDL‐C, high‐density lipoprotein cholesterol; HOMA‐IR, homeostatic model assessment—insulin resistance; hs‐TnT, high‐sensitivity troponin T; LDL‐C, low‐density lipoprotein cholesterol.

* *p* < 0.05.

The omega‐3 group showed a significant increase in serum adiponectin levels from the start to Week 8 (*p* = 0.001), while no significant change was observed in the control group (*p* > 0.05). A notable disparity in serum adiponectin levels at the start was observed between the groups, with the control group exhibiting a higher level (*p* < 0.05). Nevertheless, there was no notable disparity observed in the serum adiponectin levels between the omega‐3 and control groups when analyzed at Week 8 (*p* > 0.05) (Table 3).

Table 3 demonstrates that both the omega‐3 and control groups experienced a significant decrease in total cholesterol and LDL‐C levels from the beginning to Week 8. Statistical tests confirmed this finding (*p* < 0.001). Furthermore, there was a significant increase in serum phosphorus levels observed in both the omega‐3 and control groups from the beginning to the eighth week (*p* < 0.05). However, calcium levels showed a significant increase only in the omega‐3 group (*p* < 0.05).

There were no significant differences between the omega‐3 and control groups in baseline fasting blood glucose levels and HOMA‐IR values (*p* > 0.05). However, at Week 8, both parameters were significantly higher in the omega‐3 group compared to the control group (*p* < 0.05) (Table 3).

A significant inverse relationship was observed between serum fetuin‐A (ng/mL) concentrations measured at Week 8 and the intake of insoluble fiber (g), protein (g), protein (g/kg), animal protein (g), total fat (g), vitamin E (mg), vitamin B2 (mg), niacin (mg), phosphorus (mg), and zinc (mg) intakes of patients in the omega‐3 group (*p* < 0.05). The study also discovered a negative correlation between the levels of fetuin‐A in the blood and the intake of protein (g), protein (%), animal protein (g), and folate (mcg) intakes of patients in the control group (*p* < 0.05) (Table 4).

**TABLE 4:** The relationship between the serum fetuin‐A levels analyzed at Week 8 and the daily energy intake and the amount of intake of some nutrients.

Energy and nutrients	Omega‐3 group (*n*: 16)		Control group (*n*: 18)	
	*r*	*p*	*r*	*p*
Energy (kcal)	−0.403	0.122	−0.265	0.287
Energy (kcal/kg)	−0.404	0.120	−0.082	0.745
Carbohydrate (g)	−0.226	0.401	−0.229	0.360
Carbohydrate (%)	0.202	0.452	−0.060	0.813
Total fiber (g)	−0.470	0.066	−0.163	0.519
Soluble fiber (g)	−0.256	0.338	−0.126	0.618
Insoluble fiber (g)	−0.528	**0.036** *	−0.110	0.664
Protein (g)	−0.590	**0.016** *	−0.587	**0.017** *
Protein (g/kg)	−0.543	**0.030** *	−0.346	0.159
Protein (%)	−0.123	0.650	−0.556	**0.017** *
Animal protein (g)	−0.646	**0.007** *	−0.492	**0.038** *
Animal protein (%)	−0.161	0.552	−0.227	0.366
Vegetable protein (g)	−0.391	0.135	−0.456	0.057
Vegetable protein (%)	0.161	0.552	0.227	0.366
Total fat (g)	−0.524	**0.037** *	0.151	0.550
Total fat (%)	−0.193	0.474	0.307	0.216
SFAs (%)	0.025	0.927	0.259	0.300
MUFAs (%)	−0.208	0.439	0.377	0.123
PUFAs (%)	−0.208	0.440	0.198	0.431
Cholesterol (mg)	−0.222	0.408	−0.142	0.574
Omega‐3 fatty acids (g)	−0.118	0.663	0.161	0.524
Omega‐6 fatty acids (g)	−0.471	0.065	0.038	0.881
Omega‐6/omega‐3	−0.124	0.648	−0.077	0.762
Vitamin A (mcg)	−0.094	0.728	−0.468	0.050
Vitamin E (mg)	−0.542	**0.030** *	−0.305	0.219
Vitamin B_1_ (mg)	−0.493	0.052	−0.302	0.223
Vitamin B_2_ (mg)	−0.553	**0.026** *	−0.277	0.266
Niacin (mg)	−0.651	**0.006** *	−0.480	0.044
Vitamin B_6_ (mg)	−0.331	0.210	−0.248	0.321
Vitamin B_12_ (mcg)	0.025	0.926	0.044	0.861
Folate (mcg)	−0.192	0.475	−0.514	**0.029** *
Calcium (mg)	−0.448	0.082	−0.265	0.287
Magnesium (mg)	−0.488	0.055	0.059	0.815
Potassium (mg)	−0.300	0.259	−0.351	0.154
Phosphorus (mg)	−0.640	**0.008** *	−0.320	0.196
Iron (mg)	−0.423	0.103	−0.368	0.133
Zinc (mg)	−0.542	**0.030** *	−0.305	0.219
Copper (mg)	−0.483	0.058	−0.253	0.311

*Note:* Pearson correlation coefficient was used to examine the correlation between two values with normal distribution, while Spearman coefficient was preferred in the cases where minimum one measurement value had abnormal distribution. Bold indicates statistical significance rates were given 95% confidence interval for *p* < 0.05.

Abbreviations: MUFAs, monounsaturated fatty acids; PUFAs, polyunsaturated fatty acids; SFAs, saturated fatty acids.

* *p* < 0.05.

## 4 Discussion

Possible mechanisms underlying the anti‐atherogenic effects of omega‐3 polyunsaturated fatty acids (PUFAs) include their therapeutic impact on lipid and glucose metabolism, their positive influence on insulin sensitivity, their ability to inhibit platelet activity, and their anti‐inflammatory properties (Yanai et al. 2018). Thus, research examining the impact of omega‐3 PUFA on cardiovascular well‐being typically assesses levels of serum total cholesterol, LDL‐C, HDL‐C, triglyceride, CRP, fasting blood glucose, fasting insulin, and adiponectin levels (Jafari Salim et al. 2017; Mostowik et al. 2013). In addition to these biochemical parameters, the effects of omega‐3 PUFA intake on serum fetuin‐A level, which is associated with the risk of developing CAD, are also among the current research.

In the study of Werida et al. (2021), in which they applied 1 g/day omega‐3 supplementation to 40 hemodialysis patients for 6 months, it was determined that there was a significant increase in serum fetuin‐A levels after omega‐3 supplementation (*p* < 0.001) (Werida et al. 2021). On the contrary, in a study by Ozyazgan et al. (2013) in which they applied 1.2 g/day omega‐3 PUFA supplementation to 40 patients with Type 2 diabetes for 2 months, it was found that omega‐3 supplementation caused a decrease in serum fetuin‐A levels (*p* < 0.05) (Ozyazgan et al. 2013). Khodadustan et al. (2021), on the other hand, found that omega‐3 supplementation (2 g/day omega‐3 FA supplementations for 90 days in patients with chronic kidney failure) did not cause a significant change in serum fetuin‐A level (*p* > 0.05) (Khodadustan et al. 2021). In this study, serum fetuin‐A concentrations decreased significantly in both omega‐3 and control group from baseline to Week 8 (*p* < 0.001). In addition, while there was no significant difference between the omega‐3 and the control group in terms of baseline serum fetuin‐A levels (*p* > 0.05), serum fetuin‐A levels analyzed at Week 8 were found to be higher in the omega‐3 group (*p* = 0.001). These data indicate that medical nutrition therapy (low‐fat diet principles) administered to patients with CAD, with or without omega‐3 supplementation, results in decreases in serum fetuin‐A levels. However, it is seen that this decrease is higher only in the group that applied principles of medical nutrition therapy. This suggests that omega‐3 supplementation may reduce the decline in serum fetuin‐A levels. However, new studies on patients with CAD are needed to reach definite conclusions about the existence and direction of the relationship.

Investigating the effects of omega‐3 supplementation on the lipid profile may contribute to a deeper understanding of the relationship between omega‐3 FAs and CAD (Tadic et al. 2021). In the study of (Jafari Salim et al. 2017), in which they applied 1200 mg/day omega‐3 supplementation to half of 42 individuals with CAD for 8 weeks and placebo to the other half, it was determined that serum LDL‐C levels decreased in the omega‐3 supplementation group (*p* < 0.05) (Jafari Salim et al. 2017). In this study, it was observed that serum total cholesterol and LDL‐C levels decreased significantly in both the omega‐3 and the control groups from baseline to Week 8 (*p* < 0.001). These results suggest that medical nutrition therapy + omega‐3 FAs supplementation is not superior to medical nutrition therapy alone in terms of its positive effects on the serum lipid profile.

Individuals with hyperglycemia are at a higher risk of developing CVD compared to those with normal glucose regulation (Bilal and Pratley 2025). Therefore, while investigating the effects of omega‐3 PUFA on CVD, it is important to evaluate the effects of omega‐3 PUFA intake on glucose metabolism, insulin sensitivity and insulin resistance (Yanai et al. 2018). While some studies indicate that omega‐3 PUFAs improve insulin sensitivity (Browning et al. 2007; Juárez‐López et al. 2013), some studies have concluded that it has no effect on insulin sensitivity (Lalia et al. 2015) or reduces insulin sensitivity (Mostad et al. 2006). In the current study, there were no significant differences between the omega‐3 and the control group in terms of baseline serum fasting blood glucose levels and HOMA‐IR values (*p* > 0.05), while the parameters analyzed at Week 8 were significantly higher in the omega‐3 group compared to the control group (*p* < 0.05) (Table 3). These results indicate that medical nutrition therapy applied alone without omega‐3 supplementation in coronary artery patients may be more effective in reducing fasting blood glucose levels and HOMA‐IR value. This suggests that omega‐3 FA supplementation is not a rational approach among interventions to improve glycemic control and insulin sensitivity in patients with CAD.

There are studies evaluating the effects of omega‐3 supplementation on serum phosphorus and calcium levels (Moeinzadeh et al. 2016; Werida et al. 2021). However, these studies were mostly carried out on dialysis patients, and the results of the studies are contradictory (Moeinzadeh et al. 2016; Werida et al. 2021). In the study carried out by Werida et al. (2021) with hemodialysis patients, it was found that omega‐3 FA supplementation did not have a significant effect on serum phosphorus levels (*p* > 0.05), but decreased serum calcium levels (*p* < 0.05) (Werida et al. 2021). The results of this study indicate that omega‐3 supplementation does not have a significant effect on serum phosphorus levels, but may lead to an increase in serum calcium levels (Table 3). The effects of omega‐3 supplementation on serum phosphorus and calcium levels remain unclear due to conflicting study results in the literature (Khosroshahi et al. 2013; Liu et al. 2021; Werida et al. 2021).

Although many health benefits, including cardiovascular health, of omega‐3 PUFAs have been reported, the mechanisms underlying these effects are still under investigation (Shahidi and Ambigaipalan 2018). One of these possible mechanisms is its effects on serum adiponectin (Sepidarkish et al. 2021). In a meta‐analysis evaluating data from 52 randomized controlled studies, it was concluded that omega‐3 supplementation significantly increased serum adiponectin levels (Sepidarkish et al. 2021). In this study, serum adiponectin levels were significantly higher after 8 weeks of supplementation in the omega‐3 group in comparison to baseline (*p* < 0.05); however, the change in the control group was not significant (*p* > 0.05). In addition, while the serum adiponectin levels analyzed at baseline were found to be lower in the omega‐3 group compared to the control group (*p* < 0.05), this difference was not significant at Week 8 (*p* > 0.05). The obtained data suggest that one of the possible mechanisms underlying the positive effect of omega‐3 on cardiovascular health may be the increase in serum adiponectin levels caused by omega‐3.

Omega‐3 FA supplement is one of the important dietary supplements that have been investigated for their effectiveness in the treatment of obesity, which is known to be a risk factor for CVDs, as they have very few known side effects (Albracht‐Schulte et al. 2018). However, the effects of omega‐3 FA supplementation on body weight are unclear, and studies on the subject are conflicting (Huerta et al. 2015; Kunešová et al. 2006; Razny et al. 2015). In this study, body weight and BMI did not significantly change between baseline and Week 8 for the omega and the control group (Table 2). In the medical nutrition treatment applied to both groups, “low‐fat diet principles” were explained without any energy restriction. For this reason, it is expected that the applied medical nutrition therapy will not cause any change on these parameters. The literature and the results obtained from this study suggest that the effects of omega‐3 supplementation on body weight loss are not yet consistent and its effectiveness may vary depending on the dose/administration duration applied (Huerta et al. 2015; Kunešová et al. 2006; Razny et al. 2015).

When the pathophysiology of coronary artery disease is examined, it is seen that one of the most important modifiable risk factors for the disease is dietary habits (Bowen et al. 2018). The existence of studies revealing a relationship between nutrition and serum fetuin‐A concentrations (An et al. 2012; Icer, Koçak, Ağagündüz, et al. 2025; Icer, Koçak, Icer, et al. 2025; Icer and Yıldıran 2020b; Werida et al. 2021) necessitates questioning the relationship between CAD‐nutrition and serum fetuin‐A levels. Although we observed a positive correlation between dietary protein intake (g) and serum fetuin‐A concentrations in one of our previous studies (Icer, Koçak, Ağagündüz, et al. 2025), no significant correlation was found between these parameters in another study of ours (Icer, Koçak, Ağagündüz, et al. 2025). Yamada et al. (2015) reported that the combination of a diet with low protein and phosphate decreased the serum fetuin‐A level (Yamada et al. 2015). In this study, dietary protein (g) and animal protein (g) intakes correlated negatively with serum fetuin‐A levels analyzed at Week 8 in both the omega‐3 and the control groups (Table 4). Although these results indicate that increased dietary protein intake may have a lowering effect on serum fetuin‐A concentrations, further studies are needed to understand the existence of the relationship and whether the applied interventions affect this relationship.

It has been suggested that dietary FA intake may influence the expression of fetuin‐A (Icer, Koçak, Ağagündüz, and Yıldıran 2025; Iizuka et al. 2024). In a recent study conducted on obese patients, we found significant correlations between serum fetuin‐A levels and the dietary percentages of saturated fatty acids (SFAs) and monounsaturated fatty acids (MUFAs) (Icer, Koçak, Ağagündüz, et al. 2025). Similarly, in another study involving patients with kidney stones, we observed significant associations between serum fetuin‐A levels and dietary total fat and MUFA intake (Icer, Koçak, Icer, et al. 2025). In the current study, dietary total fat (g) intake correlated negatively with serum fetuin‐A levels analyzed at Week 8 in the omega‐3 group (*p* < 0.05). However, dietary omega‐3 FAs (g), omega‐6 FAs (g), and omega‐6/omega‐3 ratios did not have significant correlations with serum fetuin‐A levels analyzed at Week 8 in both groups (*p* > 0.05). These data obtained from the study indicate that dietary intake of omega‐3 and omega‐6 FAs (g) does not have a significant effect on serum fetuin‐A concentration.

The data obtained from this study suggest that there may be a relationship between dietary intake of vitamin E (mg), vitamin B2 (mg), niacin (mg), phosphorus (mg), zinc (mg), and folate (mcg) and serum fetuin‐A concentrations at analyzed Week 8 (Table 4). The limited number of studies in the literature (Nimptsch et al. 2015; Willis et al. 2020; Yamada et al. 2015) and some conflicting results in this study make it difficult to interpret the relationship between dietary nutrient intake and serum fetuin‐A levels. In addition, it is difficult to conclude whether the interventions applied to the groups made a significant difference in these possible relationships.

There are several limitations of our study that should be acknowledged. First, due to the open‐intervention design, both participants and researchers were aware of the intervention status, which may have introduced bias. This lack of blinding makes it more challenging to draw definitive causal inferences. Additionally, serum fetuin‐A levels can be influenced by various factors, and the study groups were not matched for all potential confounding variables. We recognize that using a regression model to adjust for other potential confounders may strengthen future studies. Ideal genetic matching was also not feasible. Furthermore, the relatively small sample size represents another limitation.

Nevertheless, our study has notable strengths. To the best of our knowledge, this is the first study to investigate the effects of omega‐3 supplementation on serum fetuin‐A levels in patients with coronary artery disease. Existing literature primarily focuses on individuals with renal failure or diabetes when exploring the relationship between omega‐3 FAs and fetuin‐A levels (An et al. 2012; Ozyazgan et al. 2013; Werida et al. 2021). Fetuin‐A levels are strongly associated with age, gender, and BMI (Eswar et al. 2024; Icer, Koçak, Ağagündüz, et al. 2025; Zhang et al. 2020). Therefore, the absence of significant differences between groups in terms of these variables is crucial for ensuring the reliability of study results. In the present study, the groups were matched for age, gender, and BMI. Consequently, the results can be interpreted independently of these potential confounding factors. Despite the limited sample size, our findings offer preliminary insights into the potential effects of omega‐3 supplementation on serum fetuin‐A levels in this specific patient population.

## 5 Conclusion

This study found that medical nutrition therapy based on low‐fat dietary principles led to a reduction in serum fetuin‐A levels in patients with coronary artery disease (CAD), regardless of omega‐3 FA supplementation. No significant correlation was observed between serum fetuin‐A levels and the amount of omega‐3 FAs consumed through supplementation and/or diet. These findings suggest that dietary modifications alone may be an effective strategy for managing serum fetuin‐A levels. While our study contributes valuable insight into the limited literature on the interaction between omega‐3 intake, nutritional status, and fetuin‐A, several limitations must be acknowledged, including the open‐label design, relatively small sample size, and the potential influence of unmeasured confounders. Future large‐scale, blinded, long‐term interventional studies are warranted to validate these findings and to further explore the potential of dietary strategies as a non‐pharmacological approach for regulating fetuin‐A levels, enhancing metabolic profiles, and reducing cardiovascular risk in at‐risk populations.

## Author Contributions

**Mehmet Arif Icer:** conceptualization (equal), data curation (equal), formal analysis (equal), investigation (equal), methodology (equal), resources (equal), software (equal), visualization (equal), writing – original draft (lead), writing – review and editing (equal). **Hilal Yıldıran:** conceptualization (equal), funding acquisition (equal), investigation (equal), methodology (equal), project administration (equal), resources (equal), supervision (equal), writing – review and editing (equal). **Asife Sahinarslan:** conceptualization (equal), investigation (equal), methodology (equal), project administration (equal), supervision (equal), writing – review and editing (equal). **Salih Topal:** conceptualization (equal), methodology (equal), writing – review and editing (equal).

## Ethics Statement

Gazi University, Faculty of Medicine Clinical Research Ethics Committee approved the study on November 26, 2020 with approval number 810. In addition, the necessary approval was obtained from the Turkish Medicines and Medical Devices Agency with the decision numbered 66175679‐514.11.01‐E.290378. The study was registered at clinicaltrials.gov (identifier: NCT05661994). All participants gave signed informed consent, and the study was conducted in accordance with the Helsinki Declaration.

## Conflicts of Interest

The authors declare no conflicts of interest.

## Data Availability

The data can be submitted from the corresponding authors upon request.
